# A Novel *CDC42* Variant with Impaired Thymopoiesis, IL-7R Signaling, PAK1 Binding, and TCR Repertoire Diversity

**DOI:** 10.1007/s10875-023-01561-0

**Published:** 2023-08-15

**Authors:** Kristian Assing, Sofie E. Jørgensen, Katrine S. Sandgaard, Kerstin De Keukeleere, Marie B.-Hansen, Mikkel S. Petersen, Ulla B. Hartling, Thanis M. K.-de Vaal, Christian Nielsen, Marianne A. Jakobsen, Eleanor Watt, Stuart Adams, Qin Hao, Christina Fagerberg, Trine H. Mogensen

**Affiliations:** 1 Department of Clinical Immunology, Odense University Hospital (OUH), Odense, Denmark; 2 Department of Biomedicine, Aarhus University (AU), Aarhus, Denmark; 3 Department of Pediatrics, Aarhus University Hospital (AUH), Aarhus, Denmark; 4 Danish Center for Neonatal Screening, Department for Congenital Disorders, Statens Serum Institut, Copenhagen, Denmark; 5 Department of Clinical Immunology, Aarhus University Hospital (AUH), Aarhus, Denmark; 6 Department of Pediatrics, Odense University Hospital (OUH), Odense, Denmark; 7 Infection, Immunity and Inflammation Section, UCL Great Ormond Street Institute of Child Health, London, UK; 8 Department of Clinical Genetics, Odense University Hospital (OUH), Odense, Denmark; 9 Department of Infectious Diseases, Aarhus University Hospital (AUH), Aarhus, Denmark

**Keywords:** CDC42, Inborn error of immunity, Mosaic, T cell receptor diversity, IL7 receptor, Lymphopenia, Newborn screening, HPV carcinogenesis

## Abstract

Genetic variants in cell division cycle 42 (CDC42) can manifest with dysmorphic features, autoinflammation, hemophagocytic lymphohistiocytosis, and thrombocytopenia, whereas defective thymopoiesis is a rare disease manifestation. We report a novel *CDC42* missense variant (c.46A > G, p.Lys16Glu) resulting in infection and HPV-driven carcinogenesis in the mosaic mother and impaired thymopoiesis and profound T cell lymphopenia in the heterozygous daughter identified through newborn screening for SCID. We found that surface expression of IL-7Rα (CD127) was decreased, consistent with reduced IL-7-induced STAT5 phosphorylation and accelerated apoptotic T cell death. Consistent with the vital role of IL-7 in regulating thymopoiesis, both patients displayed reduced T cell receptor CDR3 repertoires. Moreover, the *CDC42* variant prevented binding to the downstream effector, p21-activated kinase (PAK)1, suggesting this impaired interaction to underlie reduced IL-7Rα expression and signaling. Here, we provide the first report of severely compromised thymopoiesis and perturbed IL-7Rα signaling caused by a novel CDC42 variant and presenting with diverging clinical and immunological phenotypes in patients.

**Supplementary Information:**

The online version contains supplementary material available at 10.1007/s10875-023-01561-0.

## Introduction

Cell division cycle 42 (CDC42) belongs to the RAS superfamily of GTPases. In its active, GTP-bound, state, CDC42 regulates vital cellular processes, including adhesion, migration, proliferation, and survival as well as actin dysregulation and cytoskeletal abnormalities [10]. These functions are mediated by the interaction of CDC42 with different effector molecules, including the kinases, p21-activated kinase (PAK)1 and Wiscott–Aldrich syndrome protein (WASp), and also involve coordination of mitogen-activated protein (MAP) kinase activity, TCR signaling, and T cell homeostasis [10].

**Fig. 1:**
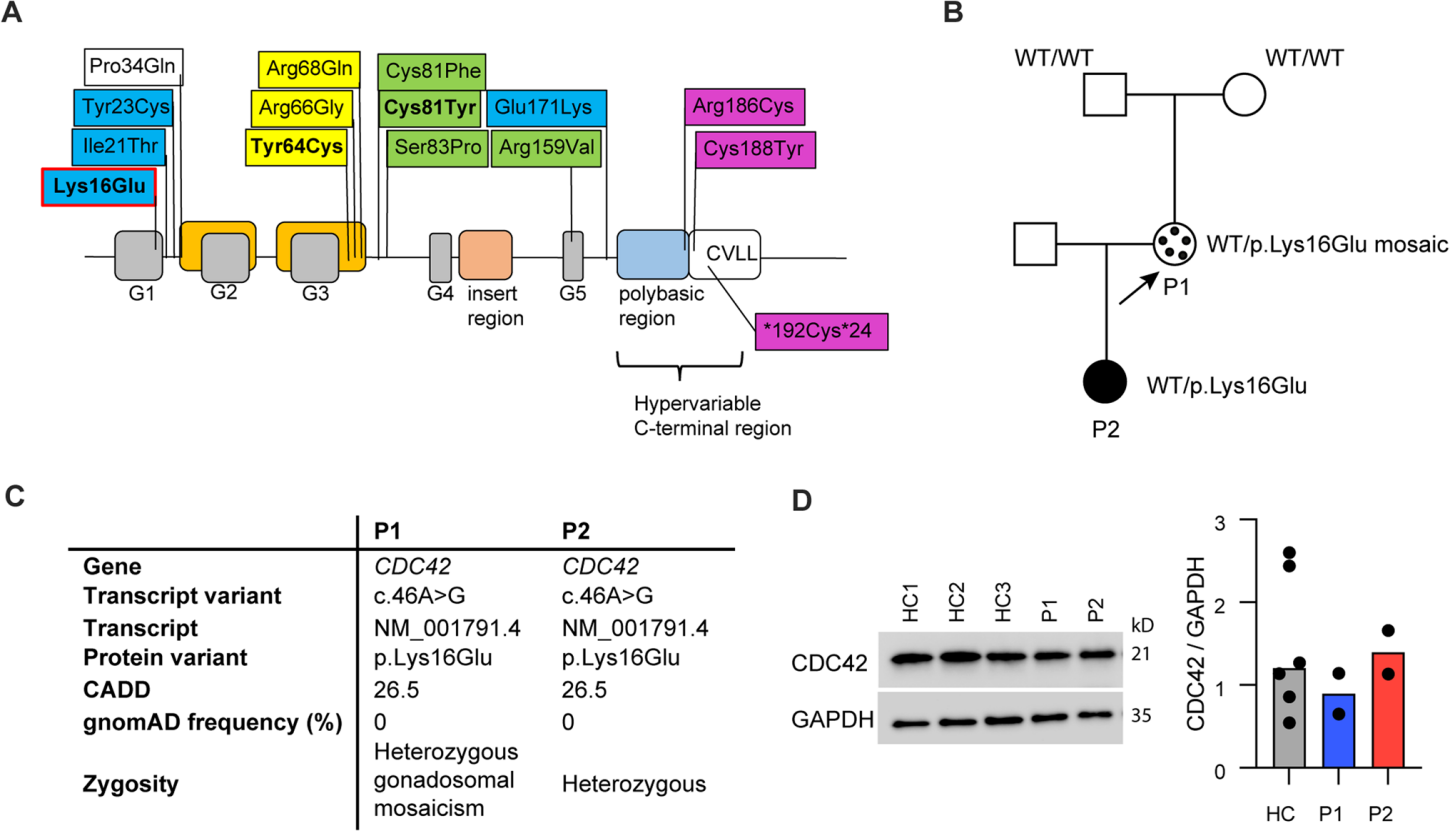
CDC42 protein structure, pedigree, and transcript information, prediction and zygosity. **A** The CDC42 p.Lys16Glu variant (red outline) results in a substitution of the positively charged lysine (position 16) for a negatively charged glutamic acid [24]. Other CDC42 variants (modified from [10]) are marked by color depending on the phenotypic group: Variants belonging to phenotypic group I are marked in yellow, group II in green, group III in blue, and group IV in purple. The variants reported to be associated with T and B cell defect are marked in bold. All variants above the protein structure are missense variants,the variant below is a read through variant. **B** Pedigree of the index patient P1 and her daughter P2. The missense variant in exon 2 of the CDC42 gene was originally identified in P2 through newborn screening and was subsequently found in her mother P1, in the latter albeit as mosaic of ~ 50% in whole blood and ~ 20% in CD3 T cells (indicated by black dots). The variant was not detected in the grandmother nor in the grandfather and was thus de novo in the mother. **C** Information on gene variant and predictions. **D** CDC42 protein expression in PBMCs from three healthy controls (HC1-3), P1 and P2, and quantification of expression levels of CDC42 relative to GAPDH in PBMCs from two independent blood draws per patient. GAPDH was used as loading control

CDC42-associated diseases are rare and due to heterozygous missense variants in relation to the five (G1–G5) functional CDC42 domains. CDC42-related phenotypes fall into four different groups, of which group I is characterized by thrombocytopenia, intellectual delay, growth retardation, and dysmorphic facial features (Takenouchi–Kosaki syndrome); group II includes developmental disorders with immunodeficiency, autoinflammation, hemophagocytic lymphohistiocytosis (HLH), and malignant lymphoproliferation; group III gives rise to Noonan syndrome (unusual facial characteristics, short stature, heart defects, and possible developmental delay); and group IV causes autoinflammation with neonatal onset pancytopenia, rash, and HLH [3, 10, 23, 24] (Fig. 1A). Among these, a few recently described variants have been associated with T and B cell defects. An 11-year-old child with a de novo p.Cys81Tyr mutation was reported to present with T cell lymphopenia, altered peripheral T cell subset distributions, including reduced fractions of recent thymic emigrants (RTE), severe B cell lymphopenia and hypogammaglobulinemia [30]. Additionally, a 19-year-old woman, with a phenotype of Takenouchi–Kosaki syndrome characterized by persistent mild thrombocytopenia, large platelet size, severe developmental delay, and characteristic facial features, due to a p.Tyr64Cys mutation, presented with declining T and B cell concentrations, reduced isotype-switched memory B cell frequencies and decreased immunoglobulin levels [21]. However, the CDC42 disease classification and the specific relation between variant location in CDC42, molecular and cellular consequences as well as somatic and immunological clinical presentation, display a high degree of complexity and remain incompletely understood.

CDC42 plays a fundamental role in T cell homeostasis by linking cytokine responsiveness, TCR signaling and apoptosis, which is critical for both protective immunity and limitation of autoimmunity. CDC42 is critical for human T cell development prior to and after rearrangement of the pre-T cell receptor [29]. Hence, dominant negative CDC42 variants are associated with increased thymocyte apoptosis and decreased thymocyte proliferation in a fetal thymus organ model [29] and Cdc42 deficient mice contain reduced numbers of single positive thymocytes [15]. Detection of signal joint T cell receptor excision circles (sjTRECs) is considered the gold standard for assessing thymic output [14], and national newborn screening programs for SCID rely on this method [1].

Here, we provide the first report of severely compromised thymic output and perturbed IL-7Rα expression and signaling, the latter secondary to impaired CDC42-PAK1 interaction, in an infant (P2) and her mother (P1), both harboring the same novel monoallelic N-terminal *CDC42* missense variant but presenting with diverging clinical and immunological phenotypes.

## Results

### Medical History of P1 and P2

Aged 29 years, P1 displays characteristic facial features with long palpebral fissures, broad nasal tip, slightly low hanging columella, short philtrum and a long neck, height (166.5 cm), and did not report learning disabilities. Early childhood pneumonias, skin infections, gastroenteritis, and urinary tract infections accompanied treatment-resistant acral warts. Following sexual debut, genital herpes virus infections and human papilloma virus (HPV)–associated *Condylomata acuminata* became clinical concerns. During her latest pregnancy (with daughter P2), HPV-associated vulva cancer with involvement of local lymph nodes was diagnosed. After primary resection, the vulva cancer relapsed and was complicated by skin and urinary tract-derived sepsis. Recurrent fevers, bleeding, HLH, or thrombocytopenia were not reported. P1 followed the national vaccination program for children including vaccination with morbilli, mumps, and rubella.

P2, the daughter of P1, was born preterm (gestational week 35 + 6). She is currently (19 months of age) thriving and appears without clear syndromic features but manifests a slight retardation in her gross motor function as she only started walking at the age of 18 months. She receives antiviral (acyclovir), antibacterial (sulfamethoxazole with trimetroprim), and antifungal (fluconazole) prophylaxis. During the first 18 months, she has experienced minor upper respiratory infections not necessitating additional antibiotic treatment. P2’s blood tests showed severe CD4 T cell lymphopenia but otherwise normal levels of total leukocytes, platelets, and immunoglobulins (except slightly elevated IgA) (Table 1). P2 was slightly anemic at 2 months (Table 1) but later normalized her hemoglobin level.

**Table 1:** Immunological characteristics of child and mother with a novel heterozygous *CDC42* variant

	Mother (P1) (29 years)	Daughter (P2) (2 months)
IgA (g/L) (95% CI) IgM(g/L) (95% CI) IgG (g/L) (95% CI) Hemoglobin (mmol/L) (95% CI) Platelets (10^9^/L) (95% CI) Leukocytes (10^9^/L) (95% CI) CD19 + (10^9^/L) (95% CI) CD4 + (10^9^/L) (95% CI) CD45RA + naïve (% CD4: mean; 90% range)^(1)^ CD45RO + memory (% CD4: mean; 90% range)^(1)^ αβ-T cell (% lymphocytes: mean; 90% range)^(1)^ γδ-T cell (% lymphocytes: mean; 90% range)^(1)^ HLA-DR + (% CD3: median; 95% CI)^(2)^ CD8 + (10^9^/L) (95% CI) CD62L + CD45RA + naïve (% CD8: mean; 90% range)^(1)^ CD62L- CD45RA + effector (% CD8: mean; 90% range)^(1)^ CD16 + /CD56 + NK cells (10^9^/L) (95% CI) CD45RA + CD31 + RTE (% CD4 +) DBSS sjTRECS (copies/10^5^ cells: median; IQR)^(3)^	**4.7** (0.7–4.3) 1.0 (0.4–2.3) **18.5** (6.1–15.7) 7.7 (7.3–9.5) **502** (165–400) 5.6 (3.5–8.8) 0.2 (0.1–0.6) 0.7 (0.3–1.7) **3%** (46%; 16–100%) **96%** (42%; 18–95%) 77% (59%; 36–98%) 8.6% (3%; 0.8–11%) **49%** (3.7%; 1.3–9.7%) **1.5** (0.2–0.9) **3%** (29%; 6–100%) 21% (19%; 7–53%) (0.0–0.6) 12.8% (6.4–51.0%)* 1742 (1800; 1263–2456)	**1.0** (0.0–0.8) (8 months of age) 0.3 (0.0–1.3) (8 months of age) 4.8 (1.3–8.2) (8 months of age) **5.8** (8.2–13.2) 493 (97–532) 13.7 (7.1–15.9) **1.9** (0.3–1.0) **0.1** (0.9–4.1) **46%** (88%; 73–100%) **46%** (13%; 4–41%) **5.8%** (66%; 48–91%) 0.3% (2%; 0.3–15%) **16%** (3.7%; 1.3–9.7%) **0.02** (0.1–0.7) **42%** (70%; 47–100%) 17% (10%; 3–40%) (0.1–1.1) **21.8%** (25.8–68.0%)* **23 and 40 (**1800; 1263–2456**)**
Follow-up	29 years	16 months
CD19 + (10^9^/L) (95% CI) CD4 + (10^9^/L) (95% CI) CD8 + (10^9^/L) (95% CI) Venous sjTRECS (copies/10^5^ cells: median: 5–95%ile)^(4)^	0.2 (0.1–0.6) (0.3–1.7) (0.2–0.9) **0–9** (227; 122–617)	0.9 (0.6–1.9) **0.3** (1.3–3.4) **0.05** (0.6–1.7) ND

*DBSS*, Dried blood spot sample; *IQR*, interquartile range; *ND*, not determined; *RTE*, recent thymic emigrants

^*^Mayo Clinic Laboratories, Rochester, USA

Values differing from the normal ranges are marked in bold

### Genetic Work-up of the CDC42 Variant, Including Assessment of Mosaicism, Maternal Engraftment, and Protein Expression

During newborn screening for SCID, P2 was diagnosed with reduced sjTRECs and marked CD4 + (0.15 × 10^9^/L; normal reference: 1.60–4.00 × 10^9^/L) and CD8 + (0.03 × 10^9^/L; normal reference: 0.56–1.70 × 10^9^/L) T cell lymphopenia, prompting whole genome sequencing of proband (P2), parents (mother (P1) and father) and maternal grandparents, supplemented by a panel of 365 PID genes. A missense variant resulting in a substitution of a positively charged lysine with a negatively charged glutamic acid (NM_001791.4:c.46A > G, p.Lys16Glu) was identified in P2 in exon 2 of the *CDC42* gene (Fig. 1A). The *CDC42* variant was also detected in P1, albeit as a mosaic, and is de novo in P1. To further examine the mosaicism of P1, whole blood was genome and exome sequenced and CD3 T cells were genome sequenced with high reading depths. Through these methods, we were able to determine that the maternal leukocyte variant allele frequency is 23.1% (read depth 2504) in the exome, corresponding to mosaicism of 46.2%. In contrast, P1’s isolated CD3 + T cell (99.9% purity) allele frequency is 9.2% (read depth 141) in the genome, corresponding to mosaicism of 18.4%. Importantly, maternal DNA was not detected in the infant DNA sample (detection limit 10–15%) (Fig. 1B). The *CDC42* variant potentially interferes with helix α1 of CDC42 which mediates binding of downstream effector molecules, including WASp- and PAK1-containing CDC42/RAC-interacting binding (CRIB) motifs [24]. The variant is not present in the genome aggregation database (gnomAD), ClinVar or Leiden open variant database (LOVD), nor is it reported in the human gene mutation database (HGMD). It is predicted clinically significant by 6 out of 6 variant prediction tools, and the combined annotation dependent depletion (CADD) score was 26.5 (Fig. 1C). No clinically significant variants in a 311 gene “cellular and adaptive immunodeficiency” and a 54 gene “immune dysregulation” panel were identified. The *CDC42* variant leads to CDC42 expression comparable to that of healthy controls as evidenced by western blotting of PBMC lysates (Fig. 1D). Collectively, this identifies a novel monoallelic potentially disease causing CDC42 variant in the infant P2. The same variant is present in the mother P1 as a germline mosaic at a fraction of ~ 50% in whole blood but only ~ 20% in T cells, thereby suggesting positive selection of WT T cells in P1.

### Signal Joint T Cell receptor Excision Circle Concentrations and Patient Immune Phenotypes

In her historic dried blood spot sample (DBSS), P1 displayed normal amounts of sjTRECs at a number of 1742 copies/10^5^ cells [1]. However, in a recent blood sample (without prior immunosuppressive treatment), P1’s sjTRECs concentrations (0–9 copies/10^5^ cells) were only 4% of normal, corresponding to > 25 times lower levels than expected for her age [22]. Recent immuno-phenotyping revealed normal B, CD4 + , and CD8 + T cell concentrations, increased fractions of activated (HLA-DR +) T cells and CD45RO + memory T cells (Table 1). P1 had protective antibody titers to tetanus toxin. P2’s DBSS consistently revealed reduced sjTRECs concentrations at 23 and 40 copies/10^5^ cells, similar to those observed in SCID (< 50 copies/1 × 10^5^ cells) [1]. Her thymus was clearly visible on a magnetic resonance scan. P2 displayed severely reduced CD4 + and CD8 + T cell concentrations, increased T cell activation (HLA-DR +), increased B-, but normal NK cell concentrations (Table 1). As for P1, P2 also exhibited markedly reduced frequencies of CD45RA + naïve CD4 + and CD8 + T cells (Table 1). Both patients had circulating γδ T cells (Table 1). Aged 16 months, P2 still displayed pronounced T cell lymphopenia and generated a protective antibody response to all 13 serotypes after a single tridecavalent pneumococcal vaccination.

### No Evidence of Dysregulated Innate Immune Responses

C-terminal variants in *CDC42* have been shown to cause severe autoinflammation with increased production of proinflammatory cytokines in response to various innate immune stimuli [11, 23]. To examine innate immune responses to PAMPs, patient and control PBMCs were stimulated by a range of Toll-like receptor agonists, which all showed normal, comparable type I interferon, and proinflammatory cytokine responses between patient and controls, thereby demonstrating the integrity of these innate signaling pathways, which is consistent with neither P1 nor P2 showing symptoms of autoinflammatory disease (supplementary Fig. 1).

### CDR3 TCR Diversity and Cluster Analysis

**Fig. 2:**
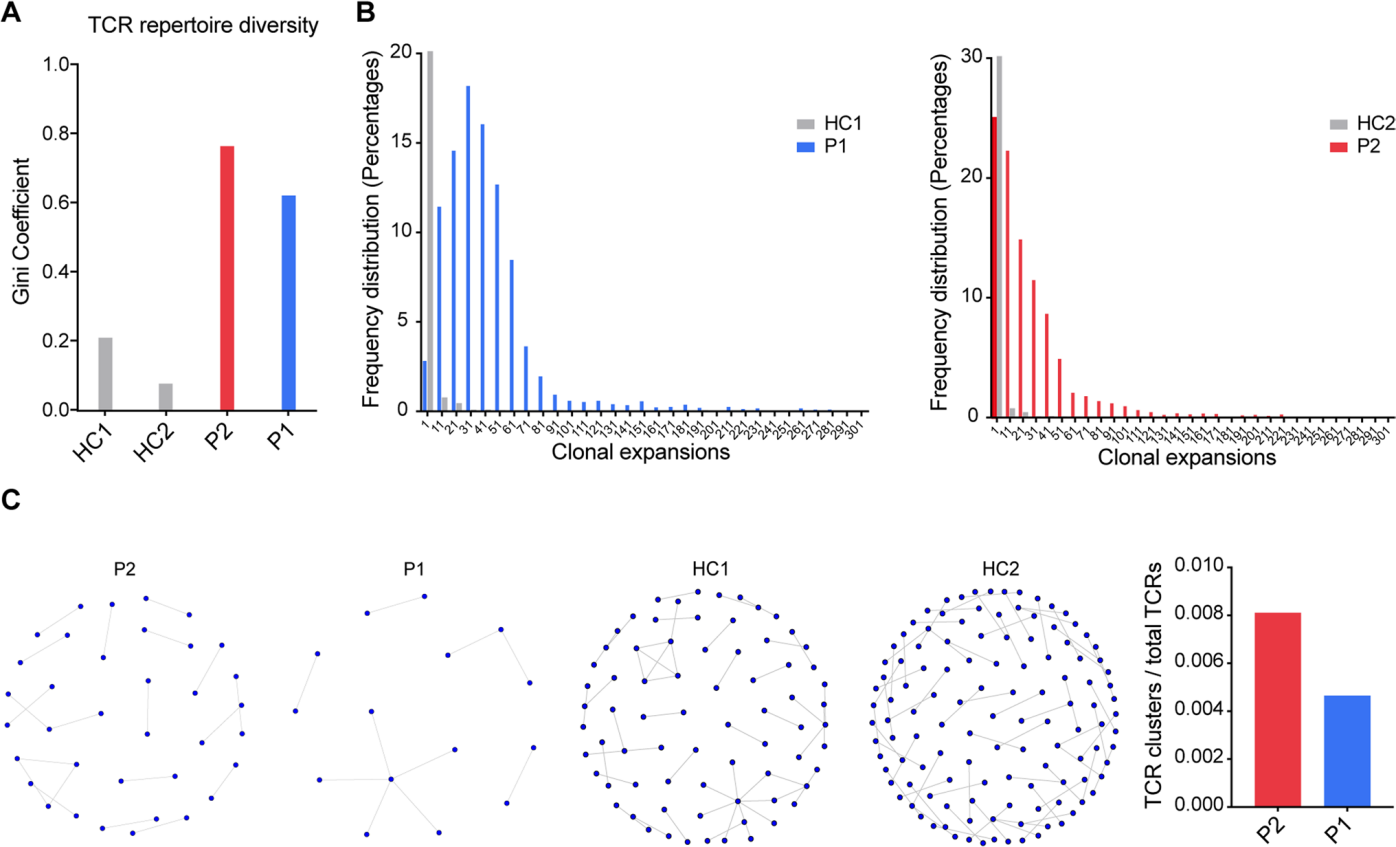
TCR CDR3 repertoire diversity and cluster formation. **A** The degree of the TCR repertoire diversity measured using the Gini coefficient in the CDR3 beta chain sequences. **B** Individual CDR3 sequence abundance distribution shown as clonal expansions. **C** TCR clusters. Structurally related CDR3s with the same motifs in their sequences forming network clusters (shown as nodes) in the T cell population and TCR clusters (nodes) as a proportion of the number of total TCRs being clustered for each sample

Evidence suggests TCR diversity to be prognostic in cervical carcinogenesis [4] and predictive for immune reconstitution [6]. We therefore ascertained CDR3 diversity. The Gini coefficients of the CDR3 beta chains in P1 and P2 were elevated (P1: 0.62, P2: 0.76) attesting to a very low TCR CDR3 repertoire diversity (Fig. 2A). The abundance distribution (Fig. 2B) showed that both P1 and P2 had reduced frequencies of unique CDR3 sequences, their frequency distributions being skewed to the right compared to those of the controls. Closely related TCRs have CDR3 regions with the same amino acid (AA) in their sequence motifs, observed and predicted to react to the same antigens [13]. To examine the presence of expanded, structurally related, TCRs in response to antigens, we examined TCR clusters (with the same motifs) across groups. CDR3 clusters from both patients were visualized as nodes in a network, each node representing a CDR3. We observed a very low number of structurally related TCR clusters in both patients (Fig. 2C), reflecting compromised antigen specific TCR repertoire diversity, which was particularly compromised in the mother (P1), likely the combined result of the CDC42 defect and an age-related phenomenon.

### Lymphocyte Proliferation, NK Cell Degranulation/Cytotoxicity, T Cell Apoptosis, and Cell Death

**Fig. 3:**
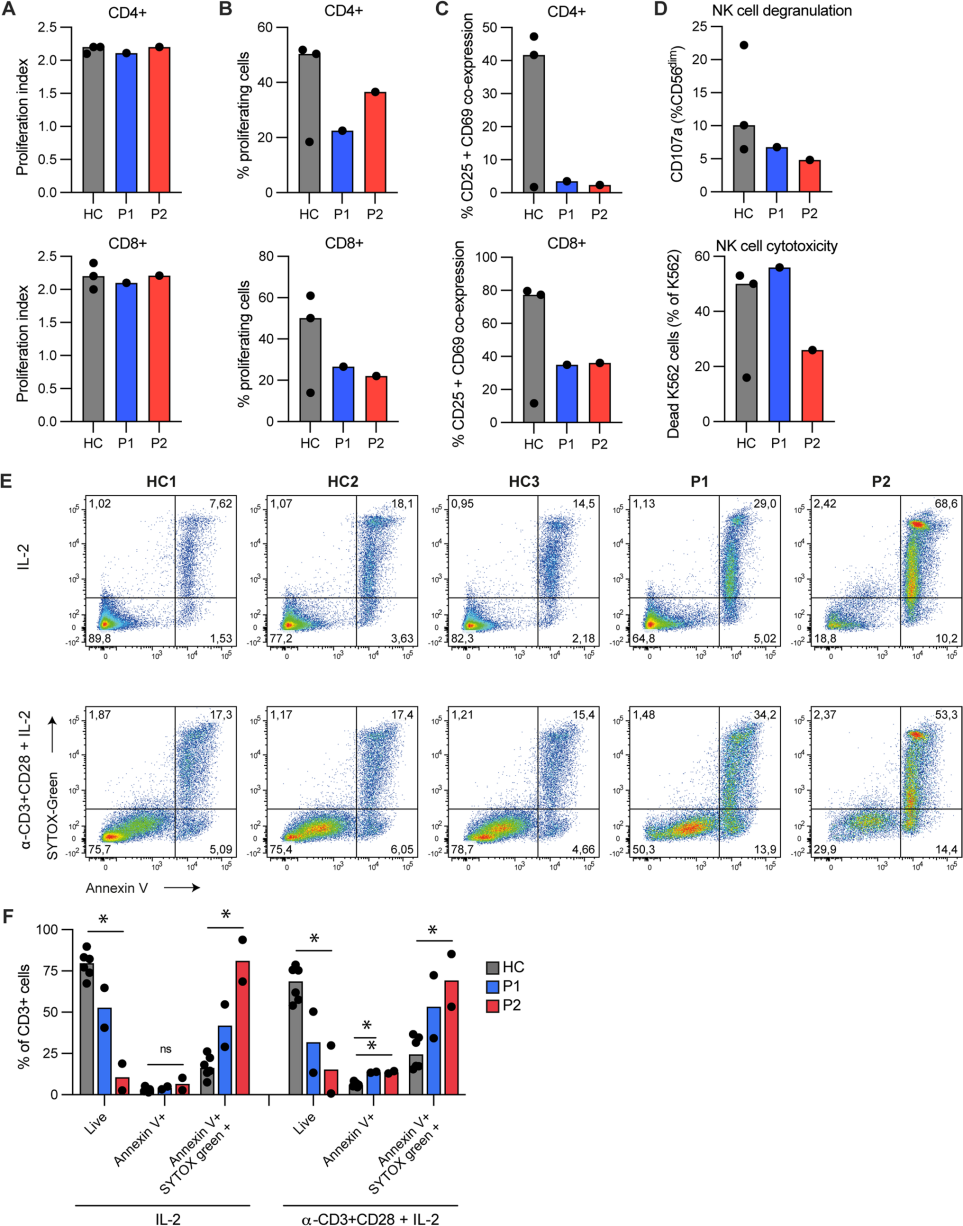
T cell proliferation, NK cell cytotoxicity, apoptosis, and cell death. **A**–**C** CD4 + and CD8 + T cells from patients (P1, P2) and healthy controls (HC) stimulated with anti-CD3/CD28 beads for 48 h. Proliferation was detected by flow cytometry. Proliferation index (number of cell divisions undergone by proliferating T cells) shown in A, frequencies (%) of proliferating T cells shown in B and CD25 CD69 co-expression shown in C. **D** NK-degranulation and NK cell–mediated cytotoxicity measured as upregulation of CD107a (lysosomalassociated membrane protein 1, LAMP1), or killing of the cell line K562, respectively. **E**–**F** Percentage of cell death (Annexin V + , SYTOX Green +) and apoptosis (Annexin V +) detected by flow cytometry in CD3 + T cells from healthy controls (HC) and patients (P1, P2) upon stimulation with IL-2 only or activated by anti-CD3/CD28 antibodies in the presence of IL-2 (α-CD3 + CD28 + IL-2). Representative individual flow plots shown in E, data pooled from two experiments performed on PBMCs isolated from different blood draws shown in F. Bars indicate median. In A–D, 3 healthy controls were used. Statistical analysis using Kruskal–Wallis test. *p < 0.05. ns, non-significant

Next T cell proliferation and death as well as NK cell activity was accessed. When stimulated with anti-CD3/CD28 beads for 48 h, CFSE stained patient CD4 + and CD8 + T cells exhibited modestly reduced percentage of proliferating cells but with a proliferation index similar to healthy controls (Fig. 3A, B) as well as reduced co-surface expression of the activation markers CD25 and CD69 (Fig. 3C). Likewise, patient NK cells demonstrated similar degranulation and K562 killing as control NK cells (Fig. 3D). Although cell viability in thawed cryopreserved PBMCs from P1 (89.5%) and P2 (87%) was comparable to that of control PBMCs (94–98%), CD3 + T cells derived from P1 and P2 showed extensive (most pronounced for P2) Annexin V + SYTOX + cell death after 72 h exposure to IL-2 (Fig. 3E, F), consistent with increased spontaneous and induced T cell death by apoptosis in P1 and P2. Activation with anti-CD3 and CD28 increased the percentage of apoptotic (Annexin V +) CD3 + T cells for P1 and P2 (Fig. 3F), with the same phenomenon observed for all donors but being most pronounced for the patients.

### IL-7Rα Surface Expression and STAT5 Phosphorylation

**Fig. 4:**
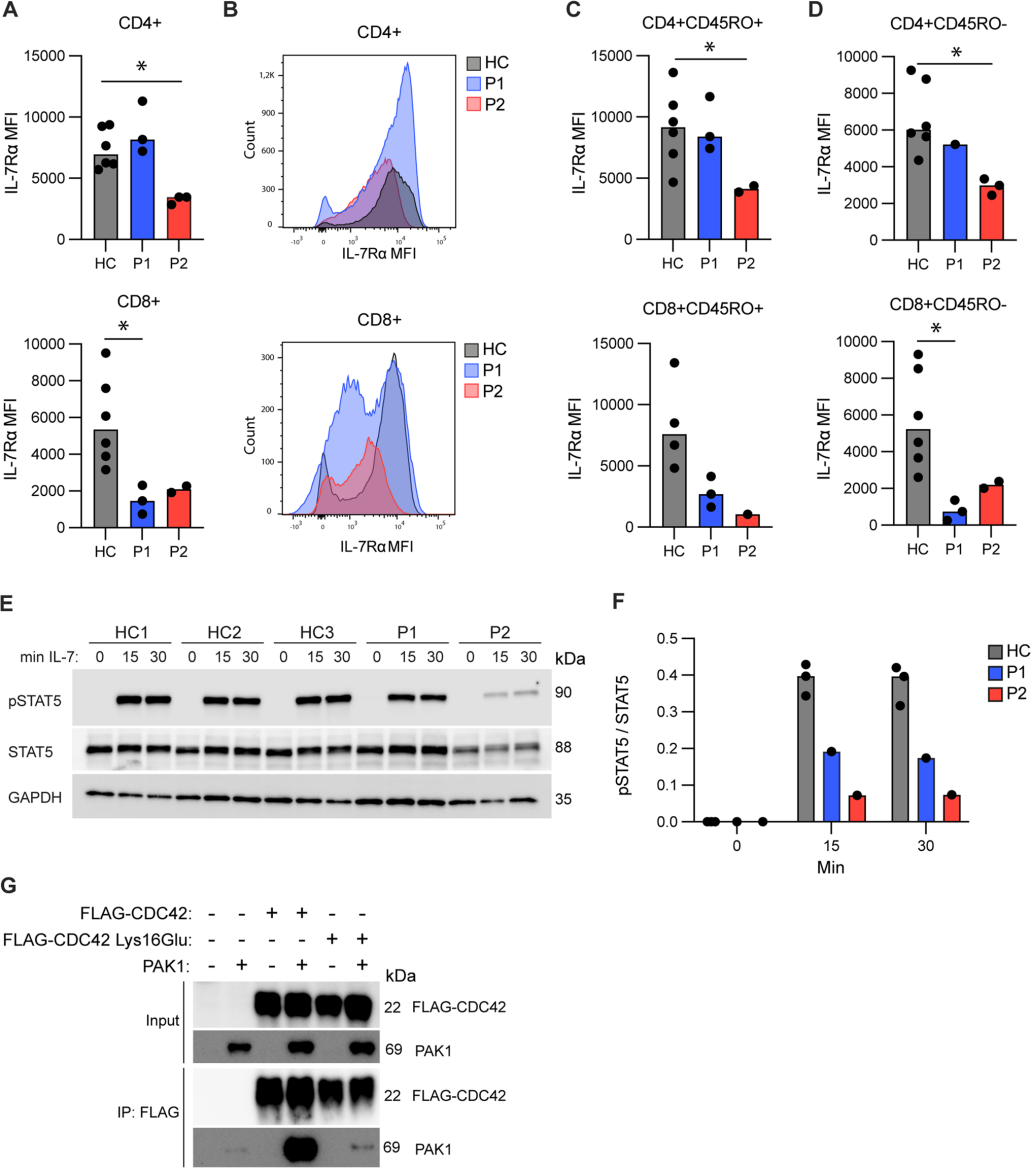
IL-7 receptor expression and signaling and CDC42-PAK1 co-immunoprecipitation. **A** Surface expression of IL-7Rα (CD127) on CD4 + and CD8 + T cells from healthy controls (HC) and patients (P1, P2). **B** Flow-histograms depicting IL-7Rα expression on CD4 + and CD8 + T cells from patients and controls. In the upper panel HC and P1 follow the same expression pattern, in the lower panel P1 and P2 follow the same expression pattern. **C,D** Surface expression of IL-7Rα (CD127) in CD45RO + memory (**C**) and CD45RO- naïve subsets (**D**), from healthy controls (HC) and patients (P1 and P2). Only measurements > 1000 events for the IL-7Rα gate are depicted. **E** PBMCs stimulated with IL-7 for 0, 15 or 30 min were blotted for induction of phosphorylated STAT5, total STAT5 and GAPDH (loading control). **F** Quantification of E depicting ratio of pSTAT5/STAT5. **G** Co-immunoprecipitation of FLAG-CDC42 and PAK1 in HEK293T cells. CDC42 Lys16Glu, CDC42 WT and PAK1 were overexpressed in HEK293T cells as shown, and co-IP was performed by pulling down FLAG-CDC42 and performing WB for the immunoprecipitated PAK1. MFI: Median Fluorescence Intensity. Bars indicate median. In panel A, C, and D, 6 healthy controls were used, except for CD8 + CD45RO + IL-7R expression where 2 controls were uninformative due to insufficient gate events (< 1000 events/gate) Statistical comparisons: Mann–Whitney *U* test (2 groups), Kruskal–Wallis test (> 2 groups). *p < 0.05, **p < 0.01

Based on previous findings that deletion of *Cdc42* in mice leads to a markedly increased expression of growth factor independence-1 (Gfi-1) and repression of IL-7Rα expression [15], we then measured IL-7Rα expression on patient cells in order to understand the mechanism underlying the disturbed T cell homeostasis. Compared to those derived from infant and adult controls, CD8 + T cells, derived from P1 and P2, displayed reduced IL-7Rα expression, whereas only P2’s CD4 + T cells demonstrated reduced IL-7Rα expression (Fig. 4A, B). This was evident on both naïve and memory cells (Fig. 4C, D). As a direct consequence hereof, PBMCs derived from P1 and P2 responded with decreased phosphorylation of the IL-7-induced transcription factor STAT5, reflected in reduced pSTAT5/STAT5 ratios after 15 and 30 min of IL-7 stimulation compared to controls. This decrease was most pronounced for P2 (Fig. 4E, F) consistent with decreased surface IL-7Rα expression affecting both her CD4 + and CD8 + T cells in contrast to only affecting CD8 + T cells in P1. These data confirm that T cells with the CDC42 variant display disturbed capacity to respond through the T cell stimulating cytokine IL-7, linking the genetic findings to the observed reduced T cell proliferation and accelerated apoptotic T cell death.

### Co-immunoprecipitation of CDC42 and PAK1

CDC42-induced responses are dependent on interaction between activated CDC42 and downstream mediators such as the kinase PAK1, and it has been demonstrated that PAK1 is necessary and sufficient for CDC42-regulated T cell homeostasis [15]. We therefore performed co-immunoprecipitation of CDC42 and PAK1, to investigate if the CDC42 Lys16Glu variant affects the interaction between CDC42 and PAK1. As expected, upon co-expression in HEK293T cells, pull-down of CDC42 WT revealed strong interaction with PAK1 (Fig. 4G). In contrast, co-expression of the CDC42 Lys16Glu variant and PAK1 resulted in significantly decreased amounts of co-immunoprecipitated PAK1, demonstrating profoundly impaired interaction between patient-derived CDC42 and PAK1 (Fig. 4G and Supplementary Figure S2). Collectively, these data demonstrate that the CDC42 variant is functionally defective in signaling downstream to one of its central substrates, the kinase PAK1, linking the CDC42 variant to defective IL-7R expression and signaling, accelerated apoptotic cell death, causing impaired thymopoiesis and T cell lymphopenia in the patients.

## Discussion

According to our knowledge, this is the first *CDC42* variant to be associated with severely compromised thymic output, markedly narrowed TCR repertories, decreased IL-7Rα expression and signaling, and abolished binding to PAK1 in humans. This is also the first report associating an N-terminal CDC42 variant with severe T cell immunodeficiency, mimicking the one encountered in T-B + SCID. The missense variant in exon 2 of *CDC42*, resulting in a substitution of a positively charged lysine with a negatively charged glutamic acid at position 16, was initially identified in P2 during newborn screening for SCID, and subsequently in the mother P1, in the latter de novo and as mosaic of 50% in peripheral leukocytes and 20% in T cells. P1’s Noonan-like features (short stature and facial characteristics) and the absence of autoinflammation, HLH and thrombocytopenia in both patients accorded with the location of this variant which, like other N-terminal CDC42 variants, was predicted to interfere with binding to downstream substrates, including PAK1 and WASp [10]. The increased infection susceptibility was evidenced by early onset bacterial and viral infections, complicated by HPV-driven vaginal carcinogenesis in (P1). In contrast, P2 has developed normally and still has an uneventful infectious history under broad antimicrobial prophylaxis. It is noteworthy, that P2, in contrast to P1, did not manifest any clear syndromic features, suggesting variable phenotypic presentation of the Lys16Glu CDC42 variant.

CDC42 governs homeostasis and apoptosis by integrating cellular signaling derived from IL-7R and TCR activation [15]. Hence, both Cdc42 deficiency in mice [15] and functionally impaired CDC42 as assessed by fetal thymus organ culture [29] led to markedly compromised thymopoiesis. In agreement with these findings, patient CD3 + T cells also demonstrated increased spontaneous and induced apoptosis and cell death, most pronounced for P2. Reconstituting *Cdc42* deficient mice with Cdc42 mutants, displaying impaired PAK1 binding, did not rescue T cell IL-7R expression, homeostasis, and survival in mice [15], thereby highlighting the essential role of preserved CDC42-PAK1 interactions for regulating these IL-7 governed processes. The IL-7R has a non-redundant role in the survival and proliferation of double negative thymocytes [32] as well as for the later immature single-positive to double receptor-positive transition [34]. Hence, attenuated IL-7R expression and signaling will negatively impact thymopoiesis and is likely one of the mechanisms underlying loss of double receptor-positive thymocytes in Cdc42 deficient mice [15]. Compared to controls, both P1 and P2 CD8 + T cells displayed reduced IL-7Rα expression, whereas for CD4 + T cells this was only the case for P2. PBMCs derived from P1 and P2 also responded with decreased STAT5 phosphorylation to IL-7 stimulation. This decrease was most pronounced for P2, consistent with decreased surface IL-7Rα expression affecting both her CD4 + and CD8 + T cells. Hence, the finding that only P2’s CD3 + T cells were globally (CD4 and CD8 subsets) affected by decreased IL-7Rα expression concords with her SCID-like sjTRECs levels at birth. Indeed, P2 phenocopied genetic IL-7Rα deficiency, the latter also leading to T-B + SCID [27]. In contrast to the experiments by Guo et al. using *Cdc42*-deficient murine T cells [15], we did not observe hyperproliferation, nor increased CD25 and CD69 expression in anti-CD3/CD28 stimulated patients T cells. This observation, conjoined with the fact that naïve patients T cell subsets also displayed markedly reduced IL-7 expression, indicates that accentuated IL-2 expression, in contrast to earlier suggestions [15], was not the major cause for our patients’ decreased IL-7Rα T cell surface expressions. Differentiated IL-7Rα expression accompanies CD8 T cell differentiation, and naïve CD8 T cells normally display uniformly high IL-7Rα expression [17, 20]. However, we observed decreased IL-7Rα expression independent of differentiation stage (both naïve and memory CD8 T cells), pointing to a different mechanism underlying aberrant IL-7Rα expression, likely related to distorted CDC42 function.

Interestingly, while P2 was born with SCID-like sjTRECs levels, P1 had normal sjTRECs levels at birth. We hypothesize that the majority of P1´s common lymphoid progenitors (CLP) carried the *CDC42* variant (reflecting her 60% leukocyte mosaicism). Due to compromised IL-7R signaling, these CLP, when entering her intrauterine/neonate thymus, would be homeostatically disadvantaged. As a consequence, preferentially thymocytes, carrying two WT *CDC42* alleles, would multiply and sustain P1’s normal sjTRECs concentrations at birth although at the price of reduced TCR diversity). Nevertheless, due to P1’s mosaic *CDC42* carrier state, increased thymocyte apoptosis likely accelerated age dependent thymic decline, leaving her with virtually extinguished sjTRECs formation at the age of 29. Contributing to P1’s current 20% peripheral CD3 + T cell mosaicism was probably death of predominantly variant positive naïve T cells, consistent with the patient’s markedly reduced fractions of naïve CD4 + and CD8 + T cells, as only naïve T cells are non-redundantly dependent upon IL-7 for homeostatic sustenance [31]. Selective death of naïve T cells will result in restricted TCR repertoires [31]. P1’s T cell IL-7Ra profile also suggested that primarily her CD8 + T cell compartment harbored the mutant *CDC42* allele. However, we cannot entirely exclude the possibility of P1 harboring the *CDC42* variant as a germline mutation with partially genetic reversal in the CD4 T cell compartment with mosaicism as the result.

IL-7 receptor signaling confers the ability of CD8 + T cell to develop into long-lived memory T cells that will retain functional responsiveness [20]. Therefore, the reduced IL-7R expression and signaling, which characterized the CD8 + T cells of P1, together with the reduced fraction of proliferating CD4 + and CD8 + T cells, accorded with impaired CD8 + T cell functionality and her susceptibility to severe recalcitrant HPV infection and development of carcinogenesis [8]. Moreover, CDC42 signaling is regulated by the guanine-nucleotide exchange factor DOCK8, defects in which are associated with IEI with increased risk of HPV-driven carcinogenesis [9]. Thus, this disease manifestation in P1 may also be at least partly driven indirectly by impaired CDC42 function in the signaling pathway regulated by DOCK8. Finally, patient and control PBMCs also responded with comparable type I IFN responses to TLR7/TLR8 and TLR9 stimulation and patient PBMCs showed no defects in IL-6 and TNF-α responses, excluding gross impairment of innate PAMP-sensing and TLR signaling in the two patients.

Consistent with the attenuated thymic function in both patients, their TCR CDR3 repertoires were markedly narrowed as reflected by elevated Gini coefficients. The abundance distribution also demonstrated that both P1 and P2 have reduced frequencies of unique CDR3 sequences compared to those of the adult controls. As the normal naïve TCR repertoire is characterized by a high proportion of unique CDR3 sequences, the abundance distributions, found in P2 (and P1), were consistent with clonal expansions of T cells with markedly reduced TCR repertoires [28]. For both patients, we observed a very low number of structurally related TCR clusters harboring the same AA in their sequence motifs, thereby suggesting a reduced capacity for generating antigen-specific polyclonal T cell responses. Impaired TCR repertoire and lack of important antigen specific TCR clusters may have contributed to early onset infections and HPV-driven vaginal carcinogenesis in P1 [4]. The reason for the TCR repertoire diversity being even more restricted in the mother P1 than in the daughter P2 may thus represent a reflection of the CDC42 defect and its influence on thymic selection together with repeated and chronic infections. It is noteworthy that despite reduced TCR diversities, P1 and P2 display residual T cell competence by generating protective antibodies to protein antigens and by controlling live attenuated vaccines (P1), thereby resembling the immunological heterogenicity evidenced by patients with genetic IL-7Rα defects [2, 35]. Interestingly, both patients have circulating γδ T cells, despite this T cell subset being dependent on IL-7R signaling [25].

Due to the clinical and immunological heterogenicity of the two patients, P2’s prognosis is difficult to ascertain. So far, she appears without major developmental delays and has remained without any severe acute or chronic infections in the context of broad antiviral, antibacterial, and antifungal prophylaxis. However, given the development of malignancy and severe infectious history of her mosaic mother, allogeneic bone marrow transplantation is considered as the treatment of choice. Clearly, the full spectrum of disease-causing variants in CDC42 and the clinical somatic, infectious, autoinflammatory, and malignant presentation of CDC42 deficiency is highly heterogeneous and remains to be fully clarified.

## Materials and Methods

### Whole Exome Sequencing and Whole Genome Sequencing

Whole exome sequencing (WES) was performed for P2, P1, the father, the maternal grandmother, and the maternal grandfather. DNA was extracted from whole blood with Maxwell® RSC Blood DNA Kit (AS1400, Promega Corporation, Madison, WI, USA), or Tecan Freedom EVO® (Tecan, Switzerland). After quantification, 1 μg of DNA was used for library preparation using NimbleGen SeqCap EZ Library SR (Roche, Basel, Switzerland). Validated libraries were pooled and paired-end sequenced on Illumina NovaSeq 6000 platform (Illumina, San Diego, CA, USA) following Illumina’s recommended protocol. Reads were mapped to the reference genome (hg19) using BWA-MEM and genotypes of targeted bases were called with GATK [26]. Variants in the coding parts of the patient’s genome are compared with relevant family members. De novo variants found in gnomAD database with frequency > 0.01, other variants with frequency > 0.03, were excluded from further analysis. The clinical significance of remaining variants was evaluated comprehensively. Besides, 311 genes associated with cellular and adaptive immunodeficiency and 54 genes associated with immune dysregulation (primary immune deficiency (PID) panels) were checked for the variants detected in both the proband and the mother, with CADD (Combined Annotation Dependent Depletion) score > 15 and population frequency in gnomAD < 0.01. Mosaicism for CDC42 in P1 was determined from fullblood with 2504 read depth (exome + genome) and from CD3 T cells with 141 read depth (repeated genome sequencing).

### Determination of Signal Joint T Cell Receptor Excision Circles

For P1, sjTREC concentrations were determined in a recent blood sample and in a birth sample. For the recent sample, DNA was extracted from whole blood, whereas the newborn screening dried blood spot sample (DBSS) was used as birth sample. The birth sample was retrieved from the Danish Neonatal screening biobank. For P2, sjTRECs were determined as part of the routine neonatal screening. DNA was extracted from the dried bloodspot samples of both P1 and P2 using the Eonis DNA extraction kit (Perkin Elmer, Turku, Finland). All samples were analyzed with the EonisPCR kit (Perkin Elmer, Turku, Finland). The kit is a multiplex RealtimePCR assay with primers and TaqMan probes for TREC and a two copy reference gene, RPP30. RealtimePCR was performed on a Quantstudio7DX (Thermo Fisher, MA, USA). TREC concentrations were calculated based on ∆Ct values between TREC and RPP30. The calculation formula is described in detail in [16]. SCID cutoffs for the Danish neonatal screening program are currently set to 50 copies per 10^5^ cells.

### Complementary Determining Region 3 T Cell Receptor Diversity and Cluster Analysis

PBMC DNA was processed as previously described [33]. In essence, DNA was amplified using a panel of primers specific to the rearranged variable, diverse, and joining (VDJ) TCR genes, encoding the hypervariable complementary determining region 3 (CDR3) beta chains. The products were size selected using Pronex beads (Promega). The products were sequenced on a MiSeq (Illumina) with an eight base pair dual indices sequence at both ends of the amplicons. Sequences for TCR CDR3 regions were annotated with reference to the International ImMunoGeneTics germline sequence to ascertain V, D, and J gene segments using Vidjil [7]. Two immunologically healthy young male adults were included in the TCR analysis as controls (C1, age 21 and C2, age 22) approved by the South Central — Hampshire A Research Ethics Committee REF number: 17/SC/0218. The distribution of TCR abundances in each CDR3 repertoire (TCR CDR3 repertoire diversity) was summarized using the Gini coefficient. The scale ranges from 0 to 1, where 0 = completely equal (x clones, all with identical frequencies) and 1 = completely unequal (i.e., tending towards sample oligo-clonality). Because the Gini coefficient is affected by total population size, CDR3 repertoires were subsampled to the same number of reads. Gini indices were computed using the ineq package (version 0.2.13) in the R environment (version 3.6.3). Minimum subsampling depth for each sample was algorithmically determined to preserve sample distribution as recently published [12]. In response to antigens, structurally related TCRs expand in clusters with identical continuous amino acids (AA) in their sequences (called motifs) [5, 13]. The presence of TCR clusters using a shared triplet metric of similarity was computed as previously described [19]. The algorithm returns clusters of TCRs with enriched motifs of three AA (presenting the TCRs as nodes in a network) constituting TCR antigen–binding motifs. The R package ggplot2 (version 2.2.1) and Prism (version 8) were used for data visualizations. All manual data analyses were executed using custom scripts written in R (version 3.6.3) and Python (version 3.7.3). The raw FastQ files are deposited at the Sequence Read Archive (https://www.ncbi.nlm.nih.gov/sra with accession to the SRA data).

### Western Blotting

PBMCs were lysed in RIPA buffer (89901, Thermo Fisher Scientific) with HALT protease/phosphatase inhibitors (78441, Thermo Fisher Scientific) and Benzonase (E1014, Sigma-Aldrich). Protein concentration was measured by Pierce BCA assay (23227, Thermo Fisher Scientific), and 5-ug protein was separated by SDS electrophoresis on 4–20% gradient gels (5671094, Bio-Rad). Proteins were transferred to PVDF membranes (1704157, Bio-Rad) using the Trans-blot Turbo transfer system. Membranes were blocked in 5% skimmed-milk PBS-T for 1 h and washed in PBS-T before incubation overnight at 4 °C with primary antibodies. Membranes were subsequently washed and incubated with secondary antibodies for 1 h at room temperature. Finally, proteins were visualized by chemiluminescence. For STAT5 westerns, membranes blotted for pSTAT5 were stripped for 10 min in Restore western blot stripping buffer (21059, Thermo Fisher Scientific), washed in PBS-T, and re-blocked in skimmed-milk before incubation with STAT5 antibody similar to above. Primary antibodies: Mouse anti-STAT5 (51–9002096, BD) 1:1000, mouse anti-STAT5 phospho (Y694) (BD) 1:1000, mouse anti-GAPDH (sc-47724, Santa Cruz Biotechnology), rabbit anti-CDC42 (2466, Cell signaling Technology) 1:1000, mouse anti-FLAG (F3165, Sigma-Aldrich) 1:1000, rabbit anti-PAK1 (2602 T, Cell signaling) 1:1000. Secondary antibodies: Donkey anti-mouse (715–036-150, Jackson ImmunoResearch) 1:1000, Donkey anti-Rabbit (711–035-152, Jackson ImmunoResearch).

### CDC42-PAK1 Co-immunoprecipitation

3 × 10^6^ HEK293T cells were seeded in 10-cm dishes and transfected with 10 ug of pCCL/PGK-FLAG-CDC42 WT, pCCL/PGK-FLAG-CDC42 K16E, and pcDNA3.1-HA-PAK1 plasmids using PEI (24765–2, Polysciences) as indicated for 24 h. Cells were lysed in Pierce IP lysis buffer (87787, Thermo Scientific) with Halt protease and phosphatase inhibitors (78441, Thermo Scientific) and 500 ug of total protein lysates were incubated with anti-FLAG magnetic agarose (A36797, Thermo Scientific) for 2 h at 4 °C while rotating. The magnetic agarose was washed twice in lysis buffer and once in water. Bound protein was eluted by 5 min incubation in Pierce IgG elution buffer (21004, Thermo Scientific) and subsequently neutralized by adding 1 M Tris–HCl pH 8.5. Total protein lysates and eluted protein samples were investigated by western blotting as described above.

### Lymphocyte Proliferation and NK Cell Degranulation and Cytotoxicity Assay

Unstained and CFSE stained PBMCs were left unstimulated or stimulated with anti-CD3/CD28 Dynabeads (Thermo Fisher, MA, USA) for 48 h according to manufacturer’s instructions. Proliferation or CD25 and CD69 surface expression for both CD4 + and CD8 + T cells derived from separate 96 well plates (2–4 wells containing 100.000 cells/well were pooled) was ascertained.

### NK Cell Degranulation and Cytotoxicity Assay

PBMCs were isolated from heparinized blood samples by density centrifugation and resuspended in complete RPMI1640 medium. Degranulation was assessed by incubating PBMCs with (and without) K562 target cells at a 1:1 ratio for 120 min. Degranulation was expressed as the percentage of CD107a positive cells among the classical NK cell population minus the corresponding percentage in the unstimulated control sample. NK cell cytotoxicity was assessed by incubating CellTrace Violet–labeled K562 target cells with (and without) PBMCs at an effector-to-target ratio of 50:1 for 24 h. The absolute count of fully viable target cells (7-AAD negative, CellTrace Violet positive) in cultures with added effector cells was compared with the corresponding number in control cultures without added effector cells. NK cytotoxicity was expressed as the median percentage of viable target cells “missing” in cultures with added effector cells (median of triplicate measurements).

### T Cell Apoptosis and Cell Death

T cell apoptosis and cell death was detected by flow cytometry as previously described [18]. Briefly, PBMCs were stimulated with either IL-2 (20 IU/mL) or a combination of anti-CD3/CD28 stimulation + IL-2 (20 IU/mL) for 72 h. PBMCs were stained for CD3 (BV605, 317322 Biolegend) and apoptosis and cell death using the APC Annexin V/Dead cell Apoptosis Kit (V35114, Invitrogen) according to the manufacturer’s instructions. Apoptotic but viable CD3 + T cells were Annexin V positive and SYTOX green negative, whereas dead or dying CD3 + T cells were Annexin V and SYTOX green positive.

### Interleukin-7 Stimulation and Phosphorylation of STAT5

Frozen PBMCs were thawed and rested overnight in RPMI1640 with 10% FBS, 1% penicillin–streptomycin. PBMCs were stimulated for 0, 15, or 30 min with 10 ng/mL human IL-7 (200–07, Peprotech). Cells were harvested, washed once with PBS and lysed in RIPA buffer for western blotting as described above.

### Cytokine Profiles in Response to PAMPs

Frozen PBMCs were thawed and rested overnight in RPMI1640 with 10% FBS, 1% penicillin–streptomycin. PBMCs were then stimulated with LPS (10 ng/ml; L2654, Sigma), R848 (1 μg/mL; tlrl-r848, InvivoGen), CpG ODN 2006 (10 μg/mL; tlrl-2006, InvivoGen) and Pam3CSK4 (200 ng/mL; tlrl-pms, InvivoGen), or left untreated for 6 h, and subsequently lysed for RNA purification. RNA was isolated using the NucleoSpin 96 RNA kit (740466.4, Macherey–Nagel) following the instructions of the manufacturer. cDNA was synthesized using iScript gDNA Clear cDNA Synthesis kit (1725035, Bio-Rad Laboratories, Inc.). Expression levels of IL-6, TNF-α, IFN-β1, IFN-α2, and TBP was measured by qPCR using TaqMan Fast Advanced Master Mix (4444964, Thermo Fischer Scientific,) and the following TaqMan probes: TBP (Hs00427620_m1), IFN-β1 (Hs01077958_s1), IFN-α2 (Hs00265051_s1), TNF (Hs01113624_g1), and IL-6 (Hs00985639_m1) (All from Thermo Fischer Scientific).

### Statistics

The Mann–Whitney *U* test was used for comparison between two variables and Kruskal–Wallis test was used for comparison between more than two variables.

### Supplementary Information

Below is the link to the electronic supplementary material.

Supplementary file1 (PDF 396 KB)

## Data Availability

All data related to this study will be shared at the request of other investigators for purposes of replicating procedures and results, according to national and international GDPR rules and following individual DTA and MTA rules with relevant investigators.
