# Supplementary material for: A Novel CDC42 Variant with Impaired Thymopoiesis, IL-7R Signaling, PAK1 Binding, and TCR Repertoire Diversity
Source: J Clin Immunol. 2023 Aug 15;43(8):1927–40. doi: 10.1007/s10875-023-01561-0 (PMC10661826; doi:10.1007/s10875-023-01561-0)
Supplement: Supplementary file 1 — Supplementary file1 (PDF 396 KB) [file 10875_2023_1561_MOESM1_ESM.pdf]

Supplementary figures

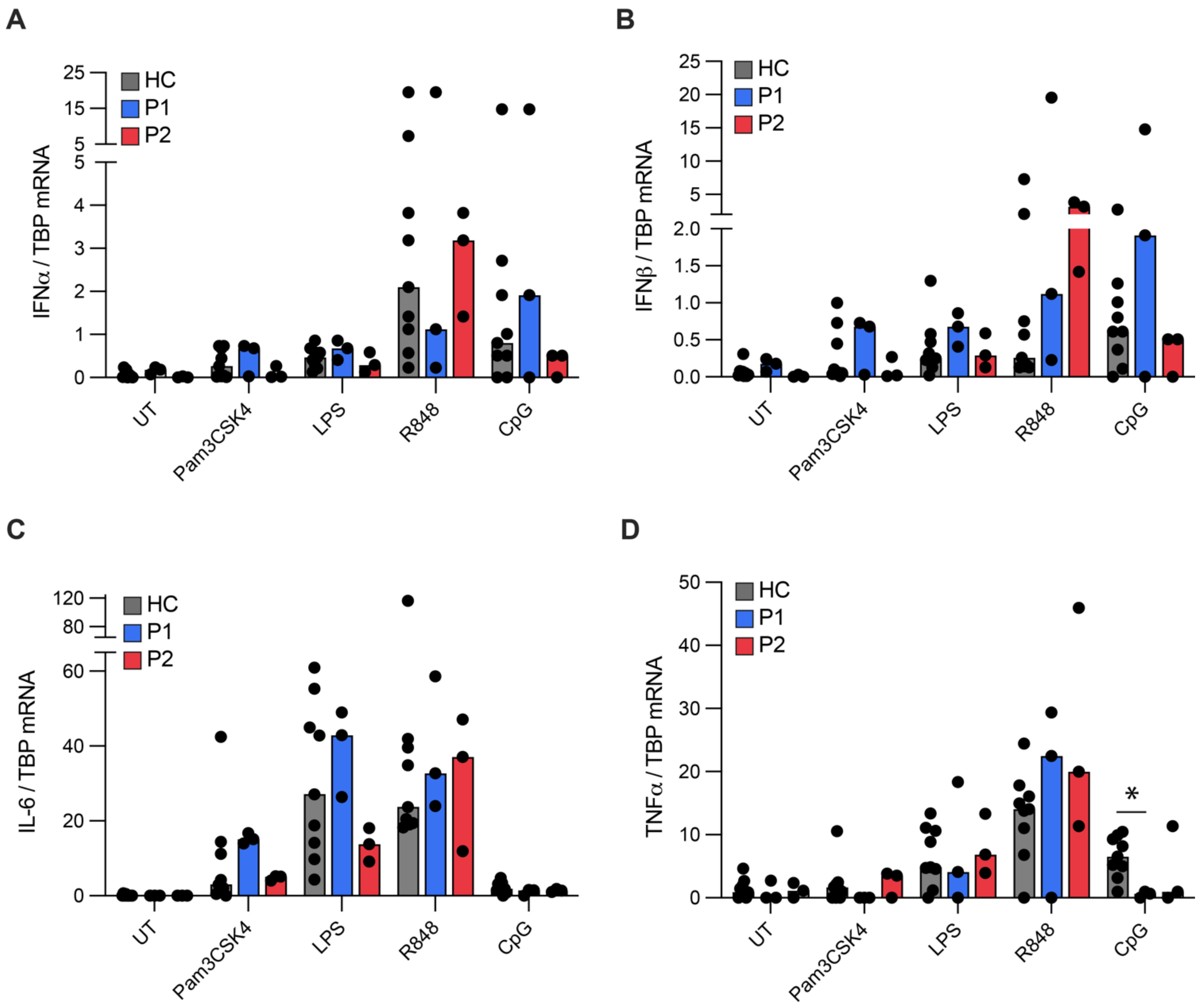

Supplementary Figure S1: PBMCs from patients (P) and healthy controls (HC) were stimulated with Pam3Csk4 (200 ng/mL), LPS (10 ng/ml), R848 (1 µg/mL), and CpG ODN 2006 (10 µg/mL) for 6 h, after which total RNA was harvested and induction of IFNα, IFNβ, IL-6 and TNFα mRNA levels were measured by RT-qPCR using TBP as reference. All stimulations were done in triplicate for each donor, HC, n=3, bars

indicate median. Statistical comparison: Kruskal-Wallis test. Only significant differences are indicated. * p<0.05.

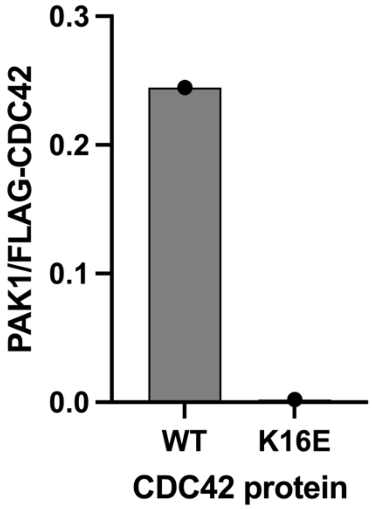

Supplementary Figure S2: Quantification of PAK1 co-IP western blot by densitometry using ImageLab software. Bands for pulled down PAK1 was normalized to bands for pulled down FLAG-CDC42 WT and FLAG-CDC42 K16E respectively.
